# The impact of vasectomy on the seminal microbiome: possible implications and source of microbes

**DOI:** 10.1093/hropen/hoag043

**Published:** 2026-05-12

**Authors:** Nerea M Molina, Inmaculada Pérez-Prieto, Celia M Tenorio, Miriam Gámiz-Aguilera, Alberto Sola-Leyva, Eduardo Salas-Espejo, Eduardo Andrés-León, Eva Vargas, Analuce Canha-Gouveia, Irene Leonés-Baños, María C Gonzalvo, Juan Fontes, José Antonio Castilla, Signe Altmäe

**Affiliations:** Department of Biochemistry and Molecular Biology I, Faculty of Sciences, University of Granada, Granada, Spain; Instituto de Investigación Biosanitaria ibs.GRANADA, Granada, Spain; Cátedra Youngner de Bioética Empírica, FiloLab-UGR, Department of Philosophy I, Faculty of Philosophy, University of Granada, Granada, Spain; Department of Biochemistry and Molecular Biology I, Faculty of Sciences, University of Granada, Granada, Spain; Department of Biochemistry and Molecular Biology I, Faculty of Sciences, University of Granada, Granada, Spain; Instituto de Investigación Biosanitaria ibs.GRANADA, Granada, Spain; Department of Biochemistry and Molecular Biology I, Faculty of Sciences, University of Granada, Granada, Spain; Instituto de Investigación Biosanitaria ibs.GRANADA, Granada, Spain; Celvia CC, Tartu, Estonia; Division of Obstetrics and Gynaecology, Department of Clinical Science, Intervention and Technology, Karolinska Institute, Huddinge, Stockholm, Sweden; Department of Gynaecology and Reproductive Medicine, Karolinska University Hospital, Huddinge, Stockholm, Sweden; Department of Biochemistry and Molecular Biology I, Faculty of Sciences, University of Granada, Granada, Spain; Instituto de Parasitología y Biomedicina ‘López-Neyra’, CSIC (IPBLN-CSIC), Granada, Spain; Instituto de Investigación Biosanitaria ibs.GRANADA, Granada, Spain; Systems Biology Unit, Department of Experimental Biology, Faculty of Experimental Sciences, University of Jaén, Jaén, Spain; Department of Biochemistry and Molecular Biology I, Faculty of Sciences, University of Granada, Granada, Spain; Instituto de Investigación Biosanitaria ibs.GRANADA, Granada, Spain; Department of Anatomy and Cell Biology, Faculty of Medicine, University of Cantabria, Santander, Spain; Department of Biochemistry and Molecular Biology I, Faculty of Sciences, University of Granada, Granada, Spain; Instituto de Investigación Biosanitaria ibs.GRANADA, Granada, Spain; Instituto de Investigación Biosanitaria ibs.GRANADA, Granada, Spain; Unidad de Reproducción, UGC de Obstetricia y Ginecología, Hospital Universitario Virgen de las Nieves, Granada, Spain; Instituto de Investigación Biosanitaria ibs.GRANADA, Granada, Spain; Instituto Avantia de Fertilidad, Granada, Spain; Instituto de Investigación Biosanitaria ibs.GRANADA, Granada, Spain; Department of Anatomy and Human Embryology, Faculty of Medicine, University of Granada, Granada, Spain; Department of Biochemistry and Molecular Biology I, Faculty of Sciences, University of Granada, Granada, Spain; Instituto de Investigación Biosanitaria ibs.GRANADA, Granada, Spain; Division of Obstetrics and Gynaecology, Department of Clinical Science, Intervention and Technology, Karolinska Institute, Huddinge, Stockholm, Sweden; Department of Gynaecology and Reproductive Medicine, Karolinska University Hospital, Huddinge, Stockholm, Sweden

**Keywords:** 16S rRNA gene sequencing, semen, urine, microbiome, microbiota, vasectomy

## Abstract

**STUDY QUESTION:**

Does vasectomy alter the seminal microbiome, and what proportions of seminal microorganisms originate from the urinary and upper reproductive tract?

**SUMMARY ANSWER:**

Vasectomy is associated with modest shifts in the seminal microbial community structure, where inter-individual variability prevails, and the semen shares over 60% of bacterial communities with urine, suggesting an influence of the urinary tract and upstream genitourinary compartments on the seminal microenvironment.

**WHAT IS KNOWN ALREADY:**

The semen harbours a polymicrobial community whose origin is not fully understood. Also, the effect of vasectomy, a common sterilization procedure, on seminal microenvironment is not clear. Recent studies with a limited sample size suggest that part of the seminal microbiome may derive from the upper genital tract, and that vasectomy may alter seminal microbial composition, potentially revealing testicular or epididymal microbial contributions.

**STUDY DESIGN, SIZE, DURATION:**

This prospective cohort study including 82 men undergoing vasectomy, with paired semen and urine samples collected before and 3 months after the procedure.

**PARTICIPANTS/MATERIALS, SETTING, METHODS:**

Paired semen and urine samples were collected pre- and post-vasectomy. The seminal and urine microbiome was analysed by sequencing the V4 hypervariable region of the 16S rRNA gene. Amplicon sequence variants were decontaminated using rigorous negative-control-based methods. Microbial diversity, taxonomic composition, and differential abundance were assessed, and functional profiles were predicted using PICRUSt2.

**MAIN RESULTS AND THE ROLE OF CHANCE:**

More than 60% of seminal bacterial genera were also present in urine, indicating substantial overlap. Vasectomy significantly altered β-diversity; however, the effect was small, and several predicted functional pathways were detected, including lipid metabolism. Although some genera differed between pre- and post-vasectomy samples, these did not remain significant after false discovery rate (FDR) correction. These findings should be interpreted considering the prevailing inter-individual variability and shared urinary–seminal taxa.

**LARGE SCALE DATA:**

The 16S rRNA gene sequencing data have been uploaded to the SRA database under BioProject ID PRJNA1355064.

**LIMITATIONS, REASONS FOR CAUTION:**

The main limitations of the study are the use of 16S rRNA gene sequencing, which limits species-level resolution, the lack of repetitive sampling, and the absence of absolute bacterial load quantification. Also, the functional pathway analyses present predictive estimates from the sequencing data rather than from direct measurements of the microbial metabolic activity.

**WIDER IMPLICATIONS OF THE FINDINGS:**

Vasectomy may induce subtle changes in the seminal microenvironment by modifying microbial composition and metabolic functions, as indicated by these exploratory analyses. Nevertheless, our study findings should not be interpreted as evidence against vasectomy, rather as novel information to better understand the dynamics of the seminal microbiome. Whether the microbial changes have any effect on the male urogenital health requires further research.

**STUDY FUNDING/COMPETING INTEREST(S):**

This work was supported by projects ENDORE (SAF2017-87526-R); Endo-Map (PID2021-12728OB-I00), and ROSY (CNS2022-135999), funded by MICIU/AEI/10.13039/501100011033 and by FEDER, EU; I.L.-B. and C.M.T. are supported by the FPU22/03045 grant and FPU23/01576, awarded by MCIN/AEI/10.13039/501100011033, respectively; additionally, S.A. obtained a mobility grant for senior researchers to do a research stay abroad funded by the Spanish Ministry of Science, Innovation and Universities (ref. PRX24/00372); I.P.-P. and A.S.-L. were supported by Becas Fundación Ramón Areces para Estudios Postdoctorales—Convocatorias XXXVI-XXXV para Ampliación de Estudios en el Extranjero en Ciencias de la Vida y de la Materia; A.S.-L. is supported by the Estonian Research Council (grant no. PSG1082); funding for open access charge: Universidad de Granada / CBUA. The authors declare no competing interests.

WHAT DOES THIS MEAN FOR PATIENTS? Vasectomy is a common and safe method of permanent contraception. However, it is not clear how this procedure affects the internal microenvironment of the semen. In our study, we examined the microbes found in the semen and urine before and after vasectomy. We found that more than half of the microorganisms in the semen also appear in urine, meaning that the majority of the bacteria are shared between these two sites. We also observed that vasectomy causes some small changes in the microbial composition of the semen, which might influence different metabolic pathways and could contribute to the alterations in the seminal microenvironment. Whether these changes have any effect on male urogenital health requires further research.

## Introduction

The human microbiota, consisting of trillions of microorganisms inhabiting various anatomical sites, has emerged as an important player in human health and disease (Rowe *et al.*, 2020). Broad research has identified the diverse microbial communities residing in the gut, oral cavity, and urogenital tract, influencing numerous physiological processes, and contributing to overall wellness (Gilbert *et al.*, 2018; Altmäe *et al.*, 2019). However, despite its significance, the exploration of the microbiome (i.e. microorganisms and their genomes) in certain human niches remains understudied. In particular, the seminal microbiome has received relatively limited attention compared to other body sites (Altmäe *et al.*, 2019).

Understanding the seminal microbiome and its origins is essential, as a growing body of research is highlighting its role in male reproductive health and disease (Altmäe *et al.*, 2019; Lundy *et al.*, 2021; Altmäe and Kullisaar, 2022; Venneri *et al.*, 2022; Suarez Arbelaez *et al.*, 2023). Recent evidence indicates that seminal microbiome dysbiosis is not only associated with altered semen parameters, including reduced motility, abnormal morphology, impaired mitochondrial function, and increased sperm DNA fragmentation, but also with a range of male reproductive disorders such as prostatitis and prostate cancer, as well as metabolic disturbances involving testosterone regulation and systemic inflammation (Weng *et al.*, 2014; Baud *et al.*, 2019; Farahani *et al.*, 2021; Garcia-Segura *et al.*, 2022; Contreras *et al.*, 2023; Zuber *et al.*, 2023; He *et al.*, 2024). Furthermore, the seminal microbiome is increasingly recognized as a determinant of couple health, given the bidirectional exchange of microorganisms between partners, and may even influence reproductive outcomes and long-term offspring health (Berard *et al.*, 2025; Cannarella *et al.*, 2025; Molina *et al.*, 2025; Qinyu *et al.*, 2025).

Vasectomy is a common procedure for sterilization, with a prevalence in Europe and North America of ∼10%, with certain countries reaching 20% among reproductive-aged men (Degraeve *et al.*, 2022; Bukavina *et al.*, 2023). This procedure causes changes in semen viscosity, pH, and prostaglandin levels that affect inflammation in addition to other functions (Brummer, 1973; Nikkanen, 1979). These oscillations in seminal characteristics could be the result of microbial alterations, as the microbiome is an important regulator of inflammation and autoimmunity (Ding *et al.*, 2020). Therefore, changes in the microbial composition following vasectomy could lead to changes in the seminal microenvironment which might have a long-term effect on men’s urogenital health (Suarez Arbelaez *et al.*, 2023).

Despite growing interest in the seminal microbiome, only a few studies have investigated the sources and acquisition pathways of microorganisms present in semen, by comparing the seminal and urinary microbiomes (Kermes *et al.*, 2003; Lundy *et al.*, 2021; Cao *et al.*, 2023). These first comparative studies on a small sample size have revealed a distinct semen microbiome with modest similarity (∼30%) to the urinary microbiome (Kermes *et al.*, 2003; Lundy *et al.*, 2021; Cao *et al.*, 2023), suggesting that the microbial composition in these fluids exhibits distinct characteristics and origin. The seminal microbiome could partly originate from the upper genital tract as the existence of microorganisms in the testis (Alfano *et al.*, 2018; Molina *et al.*, 2021), prostate (Cavarretta *et al.*, 2017; Yow *et al.*, 2017; Feng *et al.*, 2019; Jain *et al.*, 2020; Wu *et al.*, 2020), and seminal vesicles (Lei *et al.*, 2023) has been reported. However, due to the difficulty in obtaining samples from the upper genital tract, our knowledge is limited. An analysis of the seminal microbiome after the vasectomy procedure would provide complementary information of the possible routes of microbes from the upper reproductive tract. Indeed, the first results from a limited sample size highlighted alterations in the seminal microbial diversity and composition following male sterilization through vasectomy, suggesting paracrine contribution of upstream anatomic locations such as testis and epididymis as contributors to the seminal microbiome (Kiessling *et al.*, 2008; Lundy *et al.*, 2021; Suarez Arbelaez *et al.*, 2023). Nevertheless, these results were preliminary and lacking in paired urine samples and strict negative and positive controls.

In the current study, we set out to explore the seminal microbiome changes induced by vasectomy. We analyse paired seminal and urine samples collected from the same individuals before and after the sterilization procedure together with meticulous controls throughout the study protocol. The aim consists of elucidating potential sources and routes of microbial colonization from the urogenital tract, as well as investigating the effect of this common sterilization method on the seminal microenvironment.

## Materials and methods

### Study population

The study was carried out in accordance with the ethical guidelines of the Declaration of Helsinki and the legally enforced Spanish regulation, which standardizes the clinical investigation of human beings (RD 223/04). All procedures were approved by the Ethics Committee of the Investigación Biomédica de Andalucía (ref. CEIM/CEI 0463-M1-18r). Written informed consent was obtained from all subjects prior to inclusion.

A total of 82 men who were planning to undergo vasectomy were recruited at the University Hospital Virgen de las Nieves, Granada between February 2021 and October 2022. All participants donated urine and semen samples before the vasectomy and 3 months after the procedure with confirmed azoospermia in the semen analysis. In the case of presence of spermatozoa after surgery, an additional sample was collected 3 months later with confirmed azoospermia. No preoperative or postoperative antibiotics were prescribed.

Participants were requested to maintain a minimal sexual abstinence of 3–5 days before the sample collection. All semen samples were self-collected at the Hospital by masturbation into a sterile polypropylene 120-ml container (DELTALAB, Barcelona, Spain). Patients performed hand sterilization, washed the glans penis with soap and water, and collected the first urine sample (midstream urine into sterile 120-ml container) and subsequently semen sample. Semen samples were immediately provided to andrology lab technicians for processing. Before liquefaction and routine semen analysis, 200-μl aliquot from each semen sample was pipetted into a cryovial (VWR^®^, part of Avantor, Barcelona, Spain), snap-frozen in the gas phase of liquid nitrogen and stored at −80°C for further analysis.

Urine samples (3 ml) were pipetted into 1 ml of nucleic acids’ stabilizer medium (eNAT^®^ 608CS01R, COPAN Italia, Brescia, Italy). After that, the samples were kept at room temperature max 6 h (from sampling at the hospital to transfer of samples to the university laboratory). Upon arrival, samples were stored at −80°C for further analysis. BMI was calculated from the self-reported weight and height data.

### Semen analysis

The assessment of sperm parameters (i.e. sperm volume, concentration, and total progressive motility) was performed on the remaining sample in accordance with the World Health Organization guidelines from the 6th edition (World Health Organization, 2021) and the semen analysis methodology checklist (Björndahl *et al.*, 2022).

All analyses were conducted by trained andrological laboratory personnel who undergo periodic competency evaluation. The laboratory follows established internal quality control procedures and participates in an External Quality Assessment programme for semen analysis to ensure accuracy, reproducibility, and overall methodological consistency.

### DNA extraction

For microbiome analysis, genomic DNA was extracted from semen samples using the QIAamp DNA Microbiome Kit (QIAGEN, Venlo, The Netherlands) and the QIAamp UCP Pathogen Mini Kit (QIAGEN) for urine samples, following the manufacturer’s instructions. The purity, quality, and yield of the extractions were determined by measuring the A260/A280 and A260/A230 ratios with the NanoDrop ND1000 spectrophotometer (Thermo Fisher Scientific, Waltham, MA, USA). DNA concentration was quantified by fluorimetry with Qubit 4 (Thermo Fisher Scientific) and normalized.

Negative and positive controls were included and processed along with the biological samples to monitor the potential microbial contamination. Negative controls included sample collection controls for each tissue source, DNA extraction (e.g. reagent) controls, library preparation controls, and sequencing controls (Supplementary Table S1). Positive controls included the ZymoBIOMICS (Zymo Research, Irvine, CA, USA) mock community standard.

### Analysis of 16S rRNA gene sequencing

Seminal and urinary microbiomes were profiled by amplifying the V4 hypervariable region of the 16S rRNA gene and sequencing. The primers used were 515F (5′-GTGYCAGCMGCCGCGGTAA-3′) and 806R (5′-GGACTACNVGGGTWTCTAAT-3′). All PCRs were performed in 50-μl reaction volume containing 20 μl 2× Platinum Hot Start PCR Master Mix (Invitrogen, Waltham, MA, USA), 2 μl of forward primer (5 μM), 0.1 μl of reverse primer (5 μM), MilliQ lab water (Merck, Darmstadt, Germany), and extracted DNA (20 ng), under the following cycling conditions using Applied Biosystems Veriti 96-Well Thermal Cycler (Thermo Fisher Scientific): initial denaturation at 94 °C for 3 min, followed by 35 cycles of denaturation at 94 °C for 45 s, annealing at 50 °C for 1 min, and elongation at 72 °C for 90 s, with a final extension at 72 °C for 10 min. A quality control was performed using 2% agarose gel electrophoresis to verify that each sample had been amplified. The expected amplicon size was ∼380 bp. Each sample was quantitated separately by fluorimetry with Qubit 4 (Thermo Fisher Scientific) and pooled equimolarly with an optimal amount of 50 ng per sample. PCR products were first purified by column using MicroElute Cycle Pure Kit (Omega Bio-tek, Norcross, GA, USA) and next with AMPure XP magnetic beads (Beckman Coulter, Brea, CA, USA). To check the absence of primer residues and that the library size was as expected, a quality control was performed with an HS bioanalyser (Agilent Technologies, Santa Clara, CA, USA). Illumina Nextera library preparation was performed according to the manufacturer’s specifications, combining PhiX phage (20%) with the amplicon library to give diversity to the run. The final library was paired-end sequenced (2 × 300 bp) using a MiSeq Reagent Kit v.3 on the Illumina MiSeq sequencing system (Illumina, San Diego, CA, USA).

### Microbiome analysis

Raw data were demultiplexed with Illumina bcl2fastq2 Conversion Software (v2.20; Illumina, Inc.) and imported to QIIME2 software (v.2023.9; QIIME2 Development Team, Northern Arizona University, Flagstaff, AZ, USA) (Bolyen *et al.*, 2019) with a PairedEndFastqManifestPhred33 input format. Divisive Amplicon Denoising Algorithm 2 (DADA2) was used for the denoising step (Callahan *et al.*, 2016). Low-quality regions were trimmed considering a quality score below 25 to create high-quality forward and reverse reads, using the *q2-dada2* function. Taxonomy assignment of amplicon sequence variants (ASVs) was performed using the *classify-sklearn* function against the SILVA 16S v138.1 database, along with a similarity threshold of 99%. Microbial taxa were aggregated to genus level in further analysis.

The resulting ASV tables were decontaminated using the *decon()* function from the microDecon R package, with default settings (McKnight *et al.*, 2019). This approach allows to identify and remove contaminant sequences based on their proportions in negative controls, thereby eliminating contaminating reads from the biological samples (McKnight *et al.*, 2019; Molina *et al.*, 2021).

### Statistical analysis

First, we compared paired seminal and urinary microbiomes before and after the vasectomy to investigate the possible microbial contribution of the urinary tract to the seminal microbiome. Next, we compared paired pre- and post-vasectomy microbial profiles in semen samples to assess the effect of vasectomy on seminal microenvironment.

Microbiome diversity analyses were conducted in R (v.4.2.3; R Foundation for Statistical Computing, Vienna, Austria) under RStudio (v.2023.12.1 + 402; Posit Software, PBC, Boston, MA, USA) using phyloseq, vegan, microviz, and ggplot2 R packages (Wen *et al.*, 2023). Within-sample microbiome diversity (i.e. α-diversity) was estimated by Shannon diversity index and richness (i.e. number of microbial taxa), using the *diversity* and *specnumber* functions from the vegan package. Between-sample microbiome dissimilarity (i.e. β-diversity) was visualized with principal coordinate analysis (PCoA) based on the Bray–Curtis distance. For α-diversity comparisons in paired samples, Wilcoxon signed-rank test was used for significance testing with the function *wilcox.test()*. For β-diversity testing, PERMANOVA was permuted using the *adonis2* function from vegan package.

For differential abundance analysis, we applied a filter to exclude under-represented taxa, retaining only those genera with a minimum relative abundance of 0.01% in each niche. Rare species, defined as those with a zero-sum across all samples, were also removed. The analysis was conducted using the metagenomeSeq R package (Paulson *et al.*, 2013), employing a paired-sample design to compare microbial communities within individuals across multiple conditions: between semen and urine samples both pre- and post-vasectomy and between semen samples before and after vasectomy. To address variations in the number of reads across samples, the number of sequences was normalized using the Cumulative Sum Scale (CSS) method, using an algorithm that divides raw counts by the cumulative sum of counts to a percentile that captures the relatively invariant count distribution in the dataset. This method was used due to its higher sensitivity when compared to other normalization methods that measure taxon abundance (Paulson *et al.*, 2013). Finally, to identify taxon changes, normalized data were subjected to differential abundance tests based on the zero-inflated Gaussian model integrated in metagenomeSeq. All *P*-values were corrected for the multiple comparison testing applying the Benjamini–Hochberg false discovery rate (FDR) method (Benjamini *et al.*, 2006), with statistical significance set at adjusted *P*-value < 0.05.

In addition to the standard pairwise comparisons, we applied a difference-in-differences analysis to identify microbial taxa whose changes in semen after vasectomy were not solely explained by parallel shifts in urine. Specifically, we computed the contrast (Post-Semen–Pre-Semen)−(Post-Urine–Pre-Urine), which isolates the variation that is unique to the seminal microbiome by subtracting any changes also observed in urine. This approach allowed us to distinguish true vasectomy-associated alterations in semen from general fluctuations in the urogenital microbiome. In more detail, we applied a within‑subject difference‑in‑differences (DiD) design, evaluating the net change in semen after subtracting the parallel change occurring in urine from the same individual. This design inherently removes between‑subject heterogeneity and ensures that inference is driven exclusively by within‑person temporal contrasts. Metagenomic count data were processed using metagenomeSeq (Paulson *et al.*, 2013) and applying the cumulative sum scaling (CSS) to normalize library size differences and used log‑transformed CSS‑normalized abundances for downstream modelling. Low‑information taxa were filtered a priori to reduce the multiple‑testing burden. Taxon‑specific associations were modelled using the zero‑inflated Gaussian (ZIG) mixture model and the design matrix included main effects for sample type (semen vs urine) and time (post- vs pre-vasectomy), subject‑specific intercepts to account for within‑individual pairing, and a sample type × time interaction term representing the DiD estimand. Wald tests were used to assess significance, and false discovery rate was controlled using the Benjamini–Hochberg procedure. Significant interaction effects were interpreted as vasectomy‑associated compositional shifts occurring specifically in the semen and not explained by contemporaneous changes in the urine.

Phylogenetic Investigation of Communities by Reconstruction of Unobserved States (PICRUSt2, v.2.5.2; PICRUSt Development Team, Dalhousie University, Halifax, NS, Canada) was employed to predict the microbiome functional profiles (Douglas *et al.*, 2020). The ASV table was normalized by Copy Number from IMG database. Hidden State Prediction (HSP) method was used to infer gene family abundances and metagenome functional predictions were made based on the Kyoto Encyclopaedia of Genes and Genomes (KEGG) database. Principal component analysis (PCA) was performed using the *prcomp* function in R to visualize the clustering of samples based on predicted metabolic pathways. PCA was calculated based on the centred log-ratio (CLR). Differential abundance analysis was conducted using the DESeq2 R package, with FDR-adjusted *P*-values. Heatmaps were generated using the pheatmap package in *R* and *Z*-scores were calculated for each pathway in order to normalize relative abundances. For hierarchical clustering, we applied average linkage using Euclidean distance to assess the similarity between the functional profiles.

## Results

From the total of 82 men recruited into our study, the final study population comprised 56 participants with paired urine and semen samples, as certain individuals either lacked paired urine samples (n = 3), failed to attend the post-vasectomy follow-up visit (n = 15), or had samples excluded from the analysis due to technical issues such as low DNA yield and/or poor sequencing quality (n = 8). All vasectomies were uncomplicated.

**Figure 1.**
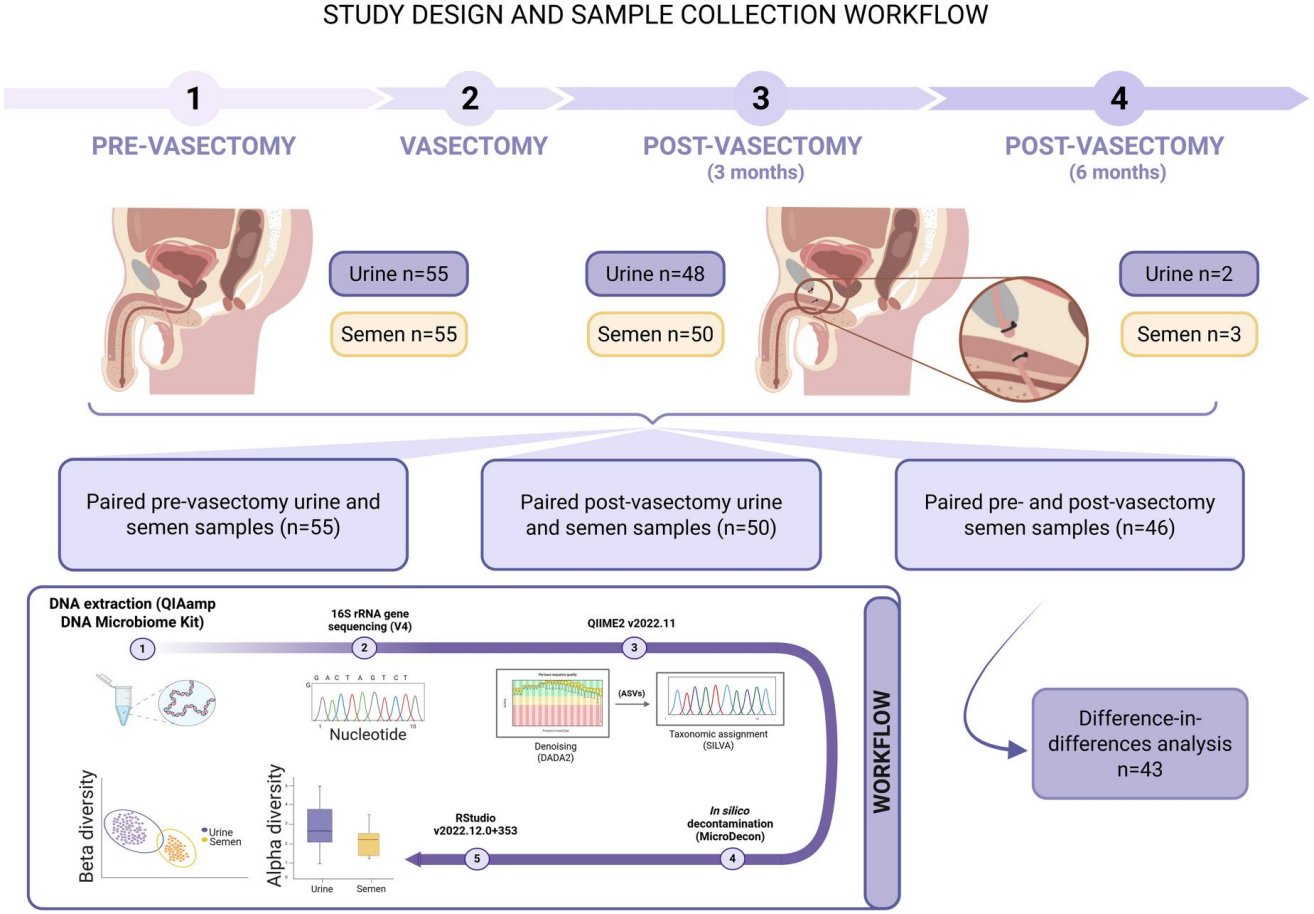
**Study design**. In total, 56 men out of 82 were included into the final setting of the study and three main analyses were performed. (**A**) Paired pre- and post-vasectomy microbial profiles in semen samples. Next analyses compared paired seminal and urinary microbiomes (**B**) before and (**C**) after the vasectomy. Created in BioRender. Altmäe, S. (2025). https://biorender.com/8rrieke.

In total, 213 samples were analysed for the microbiome composition, where 108 were semen samples and 105 were urine samples. The exact number of samples in each analysis is summarized in Fig. 1, which reflects variations due to missing paired samples, incorrect sample collection order, or the presence of spermatozoa in post-vasectomy specimens. Baseline demographics, seminal parameters, and lifestyle habits are presented in Table 1.

**Table 1.** Baseline demographics, lifestyle habits, and seminal parameters of the study participants.

Variable	Participants (N = 56)
**Age (years)**	40.3 ± 5.1
**BMI (kg/m^2^)**	25.9 ± 3.3
**Analyses**	
Pre-vasectomy semen vs urine	55 (98.2)
Post-vasectomy semen vs urine	50 (89.3)
Pre- vs post-vasectomy semen	46 (82.1)
**Smoking**	
Never	25 (44.6)
Ex-smoker	15 (26.8)
Current smoker	15 (26.8)
**Sexual dysfunction**	
No	50 (89.3)
Occasionally	5 (8.9)
**Pre-vasectomy seminal parameters**	
Abstinence (days)	4.6 ± 6.2
Volume (ml)	2 ± 1.9
Concentration (million/ml)	65.9 ± 51.9
Progressive motility (%)	47.1 ± 24
**Post-vasectomy seminal parameters**	
Abstinence (days)	3.01 ± 1.4
Volume (ml)	2 ± 1.6
**Antibiotic oral intake in the last 3 months**	
No	54 (96.4)
Yes	2 (3.6)

**Figure 2.**
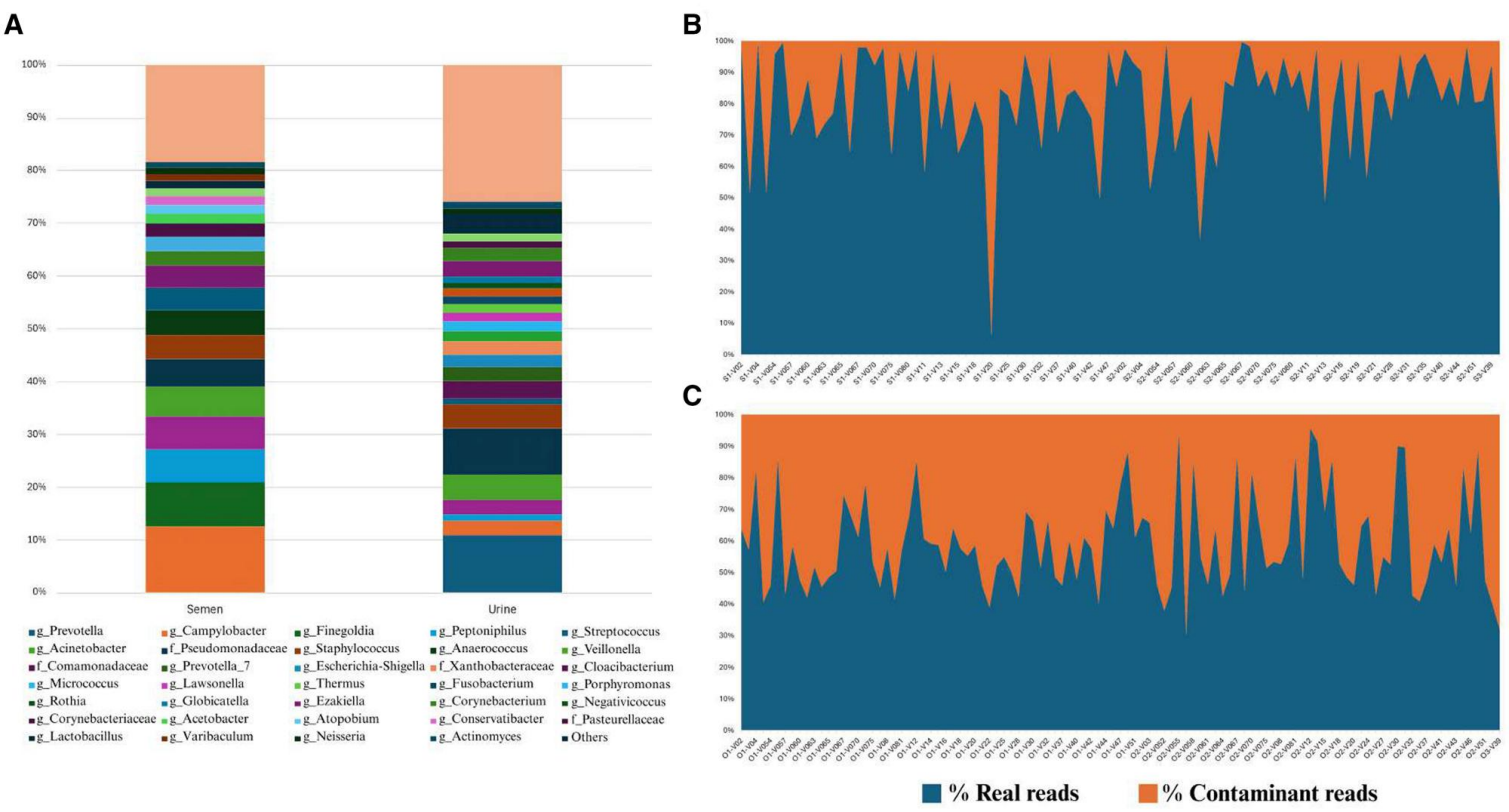
**Microbial composition and contamination assessment in semen and urine samples**. (**A**) Microbial composition in semen and urine samples. Bar charts represent those bacterial genera with a relative abundance >1%. Genera with abundance less than 1% were grouped together as “Others.” (**B**) Average percentage of reads identified as contamination after application of decontamination in semen samples. (**C**) Average percentage of reads identified as contamination after application of decontamination in urine samples.

Due to the low biomass character of the semen and urine samples, a set of negative and positive controls throughout the study protocol was used and a decontamination approach using microDecon R package was applied, which resulted in identifying 517 bacterial genera in the semen samples and 668 in urine samples (see Supplementary Table S2 for the list of contaminants, and Supplementary Table S3 for information after decontamination). The average number of reads in each site was 19 374 ± 10 771 (mean±SD) in semen samples and 31 380 ± 24 334 in urine samples. The microbial composition at the genus level in (pre-vasectomy) semen showed high proportion of *Campylobacter* (relative abundance [RA]=12.5 [median]), *Finegoldia* (RA = 8.3), *Peptoniphilus* (RA = 6.4), and *Streptococcus* (RA = 6.1), while urine presented *Prevotella* (RA = 10.9), a member of Pseudomonadaceae family (RA = 8.8), *Acinetobacter* (RA = 4.7), and *Staphylococcus* (RA = 4.7) as dominant genera (Fig. 2A; Supplementary Table S4). The average proportion of contamination in the seminal and urine samples was 19.5% (Fig. 2B) and 41% (Fig. 2C), respectively.

### Seminal versus urine microbiomes: how much is shared?

**Figure 3.**
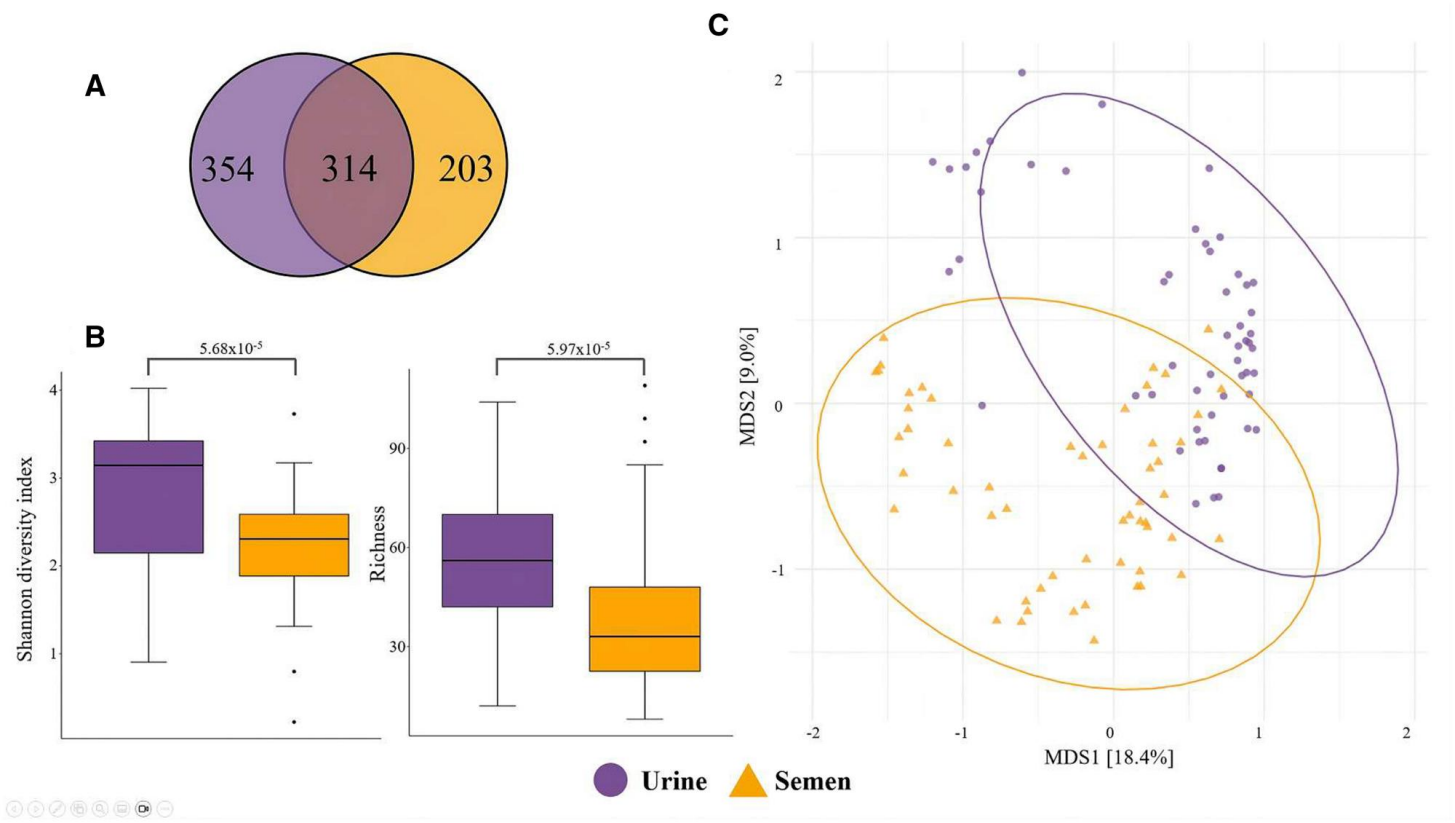
**Comparison of urinary and seminal microbiome composition and diversity**. (**A**) Venn diagram showing the distribution of identified taxa according to sample source: urine (purple), semen (orange). A total of 871 amplicon sequence variants (ASVs) were identified. Of these, 354 ASVs (53% of urine ASVs and 40.7% of all ASVs) were exclusively identified in urine samples and 203 ASVs (39.3% of semen ASVs and 23.3% of all ASVs) were unique to semen samples. The remaining 314 ASVs (36.1% of all ASVs) were shared by both niches. (**B**) α-diversity evaluated by Shannon diversity index (left) and observed richness (right). (**C**) β-diversity represented by a principal coordinate analysis (PCoA) plot based on the Bray–Curtis distance.

With the aim of unravelling the shared proportion of microbes between the two niches, urine and semen, comparative analysis of pre-operative samples was performed. A total of 871 ASVs were identified. Of these, 354 ASVs (53% of urine ASVs and 40.7% of all ASVs) were exclusively identified in urine samples and 203 ASVs (39.3% of semen ASVs and 23.3% of all ASVs) were unique to semen samples. The remaining 314 ASVs were shared by both niches, indicating that 60.7% of the ASVs present in semen were also found in urine samples (Fig. 3A; Supplementary Table S4). Urine samples revealed significantly higher α-diversity (Shannon index and observed richness Wilcoxon signed-rank *P*-value < 0.001; Fig. 3B) compared to semen samples. β-diversity analysis showed a discernible clustering between semen and urine samples (PERMANOVA, *R*^2^ = 0.092, *P*-value = 0.001; Fig. 3C). In this analysis, individuals explained 52.2% of the variance (Supplementary Fig. S1A), indicating a trend toward great inter-individual differences (*P*-value = 0.06); nevertheless, the clustering still appears to be mainly driven by sample type.

After 0.01% filtering of relative abundances, 294 bacteria were considered for differential abundance analysis between semen and urine samples. There were 33 bacteria which exhibited differential abundance between semen and urine samples. Among them, 13 significantly prevailed in semen samples, including *Varibaculum* (log-fold change [logFC]=6.39, *P*-value = 1.07 × 10^−15^) and *Anaerococcus* (logFC = 5.22, *P*-value = 1.17 × 10^−11^; Table 2). Conversely, 20 genera presented a higher proportion in urine compared to semen samples, standing out *Escherichia-Shigella* (logFC= −6.16, *P*-value = 3.30 × 10^−18^), a member of Xanthobacteraceae family (logFC= −6, *P*-value = 3.96 × 10^−19^) and *Prevotella* (logFC= −5.84, *P*-value = 1.73 × 10^−14^; Table 2).

**Table 2.** Differential abundance analysis between semen and urine samples before vasectomy.

Taxa ID	logFC	*P*-value	Adjusted *P*-value
f__Xanthobacteraceae	−6.00	1.35E−21	3.96E−19
g__Escherichia–Shigella	−6.16	2.24E−20	3.30E−18
g__Varibaculum	6.39	1.09E−17	1.07E−15
g__Prevotella	−5.84	2.36E−16	1.73E−14
g__Anaerococcus	5.22	1.98E−13	1.17E−11
g__Acetobacter	4.92	2.41E−12	1.18E−10
f__Pseudomonadaceae	−4.23	1.75E−11	7.35E−10
g__Micrococcus	−4.06	2.61E−11	9.57E−10
g__Lactobacillus	−4.12	4.68E−10	1.53E−08
g__Massilia	−4.01	7.31E−10	2.15E−08
g__Negativicoccus	4.08	1.34E−09	3.57E−08
g__Peptoniphilus	4.15	5.10E−09	1.25E−07
g__Aerosphaera	4.04	9.11E−08	1.97E−06
f__Comamonadaceae	−3.52	9.36E−08	1.97E−06
g__Actinotignum	3.85	1.34E−07	2.64E−06
g__Thermus	−3.53	2.09E−07	3.84E−06
g__Campylobacter	3.32	4.77E−07	8.25E−06
g__Cloacibacterium	−2.99	7.11E−06	0.00011618
g__Limosilactobacillus	−3.17	8.60E−06	0.00013312
g__Peptococcus	3.09	1.24E−05	0.00018212
g__Pseudoxanthomonas	−2.89	2.31E−05	0.00032321
g__Finegoldia	2.59	5.01E−05	0.00067002
g__Bacilli	−2.63	9.51E−05	0.00121516
g__Globicatella	−2.54	0.0001282	0.00157044
g__Neisseria	−2.40	0.00019514	0.00229489
g__Conservatibacter	−2.15	0.00043039	0.00486673
g__Flaviflexus	−2.35	0.00056355	0.00591731
f__Porphyromonadaceae	−2.65	0.00055957	0.00591731
g__Corynebacteriaceae	2.24	0.00099524	0.01008966
g__Geobacillus	−2.34	0.00154459	0.01513694
g__Atopobium	1.94	0.00240618	0.02281986
g__Ezakiella	2.12	0.00312723	0.02873142
g__Staphylococcus	−1.82	0.00559869	0.0498792

Microbial taxa with a relative abundance >0.01% were compared between groups using metagenomeSeq.

The sub-analysis of the seminal versus urine microbiomes in post-vasectomy samples reflected similar results, where 71.8% of the microbes present in semen were also found in urine samples (Supplementary Fig. S2A; Supplementary Table S5). Urine samples revealed significantly higher α-diversity (Shannon index and observed richness Wilcoxon signed-rank *P*-value < 0.001; Supplementary Fig. S2B) and β-diversity analysis confirmed a significant dissimilarity between semen and urine samples (PERMANOVA, R^2^=0.116, *P*-value = 0.001; Supplementary Fig. S2C). Remarkably, individuals explained 56.3% of the variance (*P*-value = 0.001; Supplementary Fig. S1B), which means that inter-individual differences are greater than the differences between sample types. Also, the differential abundance analysis between semen and urine samples resulted in similar results, where *Anaerococcus* (logFC = 6.95, *P*-value = 1.07×10^−35^), *Varibaculum* (logFC = 6.54, *P*-value= 4.91×10^−22^), and *Peptoniphilus* (logFC = 5.02, *P*-value = 2.87×10^−16^) were more prevalent in semen while a member of the Xanthobacteraceae family (logFC= −5.87, *P*-value = 3.59×10^−21^) prevailed in urine (Supplementary Table S6).

### The effect of vasectomy on seminal microbiome

**Figure 4.**
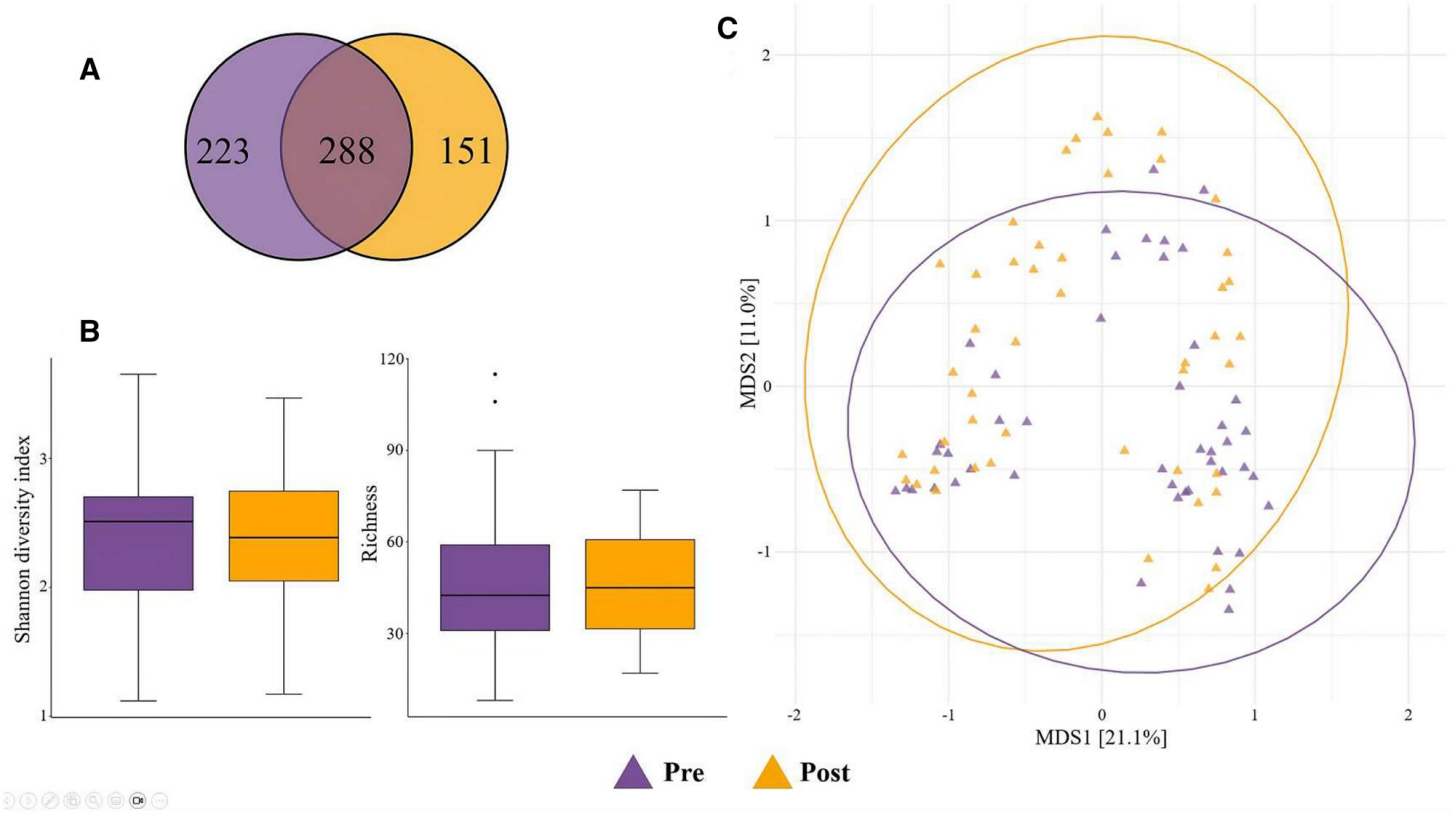
**Comparison of pre- and post-vasectomy seminal microbiome composition and diversity**. (**A**) Venn diagram showing the distribution of identified taxa in semen according to sample source: pre- (purple), post-vasectomy (orange). A total of 662 amplicon sequence variants (ASVs) were identified. Of these, 223 ASVs (43.6% of pre-vasectomy ASVs and 33.7% of all ASVs) were exclusively found in pre-vasectomy semen samples and 151 ASVs (34.3% of post-vasectomy ASVs and 22.8% of all ASVs) were unique to post-vasectomy samples. (**B**) α-diversity evaluated by Shannon diversity index (left) and observed richness (right). (**C**) β-diversity represented by a principal coordinate analysis (PCoA) plot based on the Bray–Curtis distance.

The analysis of pre- and post-vasectomy semen samples revealed 662 unique ASVs, with 223 ASVs exclusive to pre-vasectomy samples and 151 ASVs unique to post-vasectomy samples (Fig. 4A; Supplementary Table S7). While α-diversity showed no statistically significant changes (Shannon index [Wilcoxon signed-rank *P*-value = 0.8881] and observed richness [Wilcoxon signed-rank *P*-value = 0.8833]; Fig. 4B), β-diversity indicated a significant microbial dissimilarity between pre- and post-vasectomy samples (PERMANOVA, *R*^2^ = 0.025, *P*-value = 0.007; Fig. 4C). Individuals explained 67.9% of the variance (*P*-value = 0.001; Supplementary Fig. S1C).

For differential abundance analysis, 223 bacteria presented a relative abundance >0.01%, where a member of the Corynebacteriaceae family (logFC = 1.78, *P*-value = 0.024) revealed higher proportion in pre-vasectomy samples, while *Porphyromonas* (logFC=−2.12, *P*-value = 0.005), *Peptoniphilus* (logFC=−1.75, *P*-value = 0.008), *Varibaculum* (logFC=−1.67, *P*-value = 0.024 < 0.05), and *Anaerococcus* (logFC=−1.53, *P*-value = 0.025) showed increased abundance in post-vasectomy samples (Table 3).

**Table 3.** Differential abundance analysis between pre- and post-vasectomy semen samples.

Taxa ID	logFC	*P*-value	Adjusted *P*-value
g__Porphyromonas	−2.12	2.23E−05	0.0049795
g__Peptoniphilus	−1.75	7.23E−05	0.00805868
g__Varibaculum	−1.67	0.00036263	0.02434123
g__Corynebacteriaceae	1.78	0.00043661	0.02434123
g__Anaerococcus	−1.53	0.00056411	0.02515934
g__Acinetobacter	1.74	0.00199932	0.07430815
g__Lactobacillus	−1.43	0.00295758	0.09422016
f__Weeksellaceae	1.50	0.00393816	0.10055763
f__Aerococcaceae	−1.57	0.00433819	0.10055763
f__Pseudomonadaceae	1.44	0.00450931	0.10055763
g__Fastidiosipila	−1.52	0.00574352	0.10673381
g__Finegoldia	−1.24	0.00566695	0.10673381
g__Ezakiella	−1.43	0.00828239	0.14207488
g__Fenollaria	−1.36	0.0157919	0.23584803
g__Conservatibacter	1.24	0.01586422	0.23584803
g__Ameyamaea	1.24	0.01919244	0.26749458
g__Dialister	−1.21	0.02301153	0.28508727
g__Bosea	1.33	0.02285478	0.28508727
g__Corynebacterium	−0.99	0.02745387	0.32222174
g__Fusobacterium	1.07	0.03212066	0.3581454
g__Negativicoccus	−1.03	0.03657442	0.38838549

Microbial taxa with a relative abundance >0.01% were compared between groups using metagenomeSeq.

To identify microbial taxa whose differential abundance in semen before and after vasectomy was not attributable to the urinary microbiome, a double comparative analysis using paired pre- and post-vasectomy samples from a subset of participants was performed (each contributing four matched samples: pre- and post-vasectomy semen and urine). This analysis revealed *Globicatella* (logFC = 2.44), *Anaerococcus* (logFC = 2.23), and *Escherichia–Shigella* (logFC = 2.02), showing a relative increase in semen following vasectomy. None of these genera passed the FDR-adjusted significance threshold (Supplementary Table S8).

**Figure 5.**
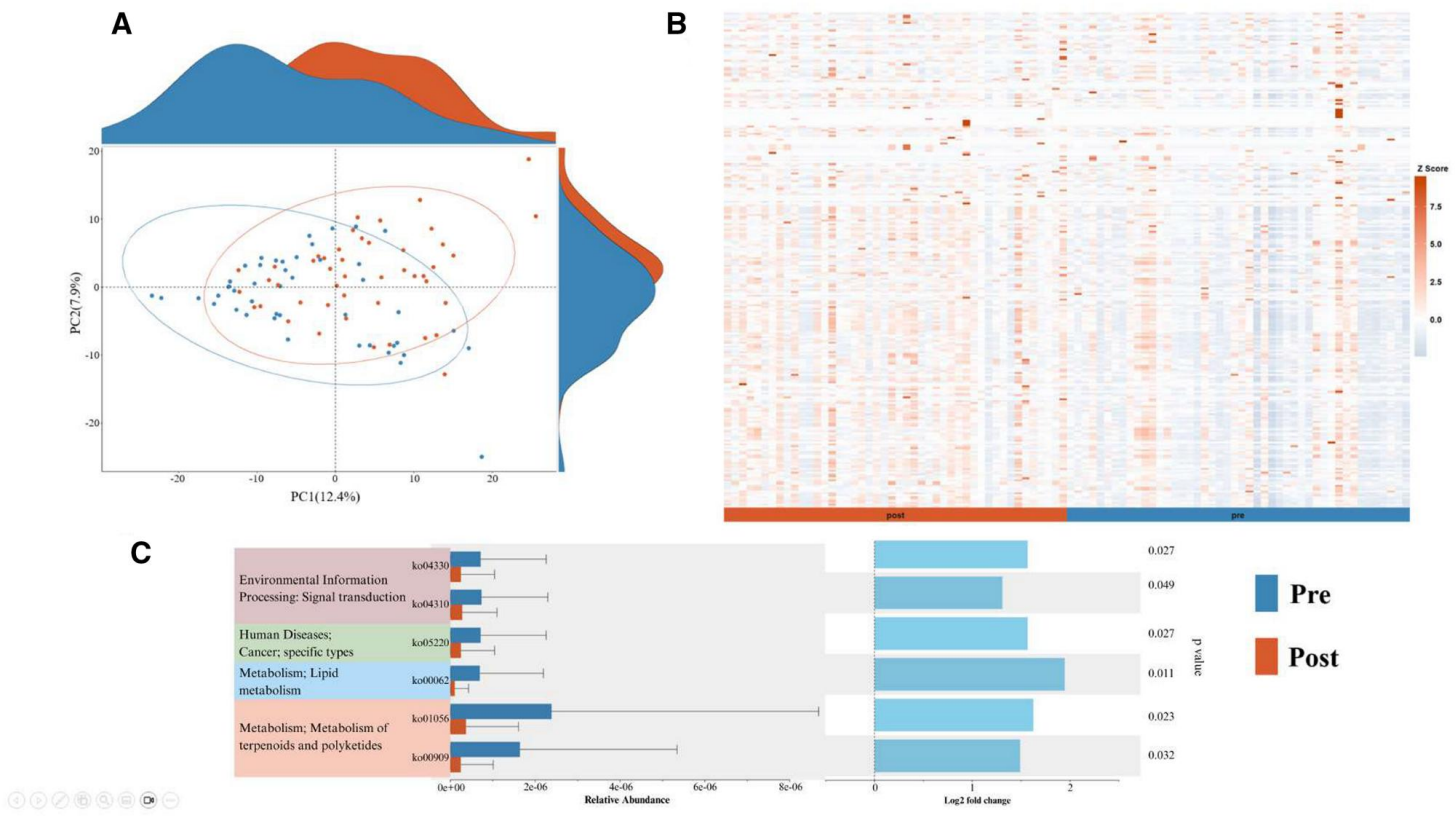
**Functional prediction analysis of seminal microbiome before and after vasectomy**. (**A**) Principal Coordinate Analysis (PCA) of predicted metabolic pathways based on the centred log-ratio (CLR). The density plots in the top and right margins show the distribution of principal component values for the pre- and post-vasectomy samples, respectively. The correlation between the original variables and the principal components was evaluated using correlation coefficient of Pearson. (**B**) Heatmap of significantly different functional profiles inferred by PICRUSt2. The heatmap provides an overview of predicted metabolic pathways across individual samples, allowing visual assessment of clustering pattern and inter-individual variability between pre- (blue) and post-vasectomy (red) samples. Each row represents a metabolic pathway, and each column represents an individual sample. The colours indicate *Z*-score values, with red representing high relative abundance and blue representing low relative abundance. *Z*-scores were calculated by normalizing the relative abundances of metabolic pathways to highlight significant differences between samples. Hierarchical clustering was generated using the hierarchical clustering algorithm with Euclidean distance as the metric and average linkage method. (**C**) Prediction of differentially abundant metabolic KEGG pathways regarding pre- and post-vasectomy. The plot shows the significantly differentially abundant metabolic pathways in terms of relative abundance between pre- (blue) and post- (red) vasectomy. Error bars represent the 95% CI, calculated from the SD of the relative abundance. The *y*-axis shows the categories of metabolic pathways according to the Kyoto Encyclopedia of Genes and Genomes (KEGG) database, while the *x*-axis shows the relative abundance of these pathways. The horizontal bars on the right in blue represent the log2-fold change for each pathway, with the associated *P*-values shown to the right of the plot, obtained using the Student’s *t*-test adjusted by the Benjamini–Hochberg method to control the false discovery rate (FDR).

The functional characteristics of the microbial communities in semen samples collected before and after vasectomy procedures exhibited a partial segregation between the two niches. Although the density PCA plot showed overlapping of the samples, there were differences in the distribution illustrated by the peaks with opposite behaviour, suggesting a trend of different functions in both niches (Fig. 5A). A heatmap was generated to visualize the relative abundance of predicted metabolic pathways in pre- and post-vasectomy samples (Fig. 5B). The hierarchical clustering indicated distinct patterns in the functional profiles of the microbial communities, with significant reduction in signal transduction (ko04330) (logFC = 1.54, *P*-value = 0.027) and (ko04310) (logFC = 1.36, *P*-value = 0.049), specific types of cancer (ko05220) (logFC = 1.57, *P*-value = 0.027), lipid metabolism (ko0062) (logFC = 1.91, *P*-value = 0.011), metabolism of terpenoids and polyketides (ko01056) (logFC = 1.65, *P*-value = 0.023), and other compound metabolism (ko00909) (logFC = 1.46, *P*-value = 0.032) after the vasectomy procedure (Fig. 5C).

## Discussion

In the current study, we describe and compare the semen and urine microbiomes in paired samples from the same individuals before and after the vasectomy to better understand the composition and dynamics of the seminal microenvironment. Our results indicate that the semen shares over 60% of bacterial communities with urine and that the vasectomy procedure is associated with modest changes in the seminal microbial composition, altogether suggesting the potential influence of upstream anatomical compartments (testis and epididymis) on seminal microenvironment.

The most abundant microbial taxa detected in semen were *Campylobacter, Finegoldia, Peptoniphilus*, and *Streptococcus*, while urine was dominated by *Prevotella, Acinetobacter, Staphylococcus*, and members of the family Pseudomonadaceae. All these taxa have been reported in healthy individuals in both semen and urine, except *Acinetobacter*, which is considered an opportunistic pathogen (Aragón *et al.*, 2018; Lundy *et al.*, 2021; Bukavina *et al.*, 2023; Chatzokou *et al.*, 2025; Zakaria *et al.*, 2025). Regarding specific genera, semen displayed higher abundances of *Varibaculum* and *Anaerococcus*, and reduced *Prevotella and Escherichia–Shigella*, among others, in comparison with urine. All these genera have been previously described in both niches (Lundy *et al.*, 2021; Cao *et al.*, 2023).

Despite sharing a large fraction of microbes between the urine and semen, the α-diversity analysis demonstrated differences between the two sample types, with urine exhibiting higher microbial diversity. Previous studies have obtained contrary results, detecting higher α-diversity in semen (Lundy *et al.*, 2021) or no differences in microbial α-diversity between semen and urine (Cao *et al.*, 2023). These differences could arise from small sample size analysed in previous studies (Kiessling *et al.*, 2008; Lundy *et al.*, 2021; Suarez Arbelaez *et al.*, 2023), or different sampling order (Cao *et al.*, 2023). Also, β-diversity analysis revealed distinct clustering patterns between the paired semen and urine samples, indicating unique and separate microbial communities in each sample type. These findings are consistent with previous studies (Lundy *et al.*, 2021; Cao *et al.*, 2023). The differences in microbial communities between the semen and urine samples were consistently smaller within the same individual than in the same sample type between different individuals, suggesting that each man retains a characteristic “urogenital microbial fingerprint” that spans different tissue types. The concept of personalized microbiome along the reproductive tract has been suggested also in previous studies (Franzosa *et al.*, 2015; Canha-Gouveia *et al.*, 2023).

The high similarity between the seminal and urine microbiomes in our study (>60%), contrary to the previous studies that indicate ∼30% (Lundy *et al.*, 2021), is expected due to the anatomical proximity of the urethra and the ejaculatory ducts. Bacterial cross-“contamination” could occur during urination or ejaculation due to their common exit pathway from the body. Another possible explanation could be related to biofilm formation. Many of these genera are known for their biofilm-forming capabilities (Brook, 2007; Turroni *et al.*, 2014; de Souza *et al.*, 2015; Wang *et al.*, 2023; Zafer *et al.*, 2024), which could allow them to persist in the genitourinary tract, colonize both the urinary and reproductive systems, and possibly influence the microbiome composition of both niches. Another important aspect is that previous studies comparing urine and seminal microbiomes did not control for bacterial contaminants, which in our study represented 20% of all detected microbes in semen and 40% in urine. Nonetheless, the mechanisms behind the microbial differences in semen and urine, as well as its potential effects on sperm quality, require further investigation.

When comparing the effect of vasectomy on seminal microbial composition, five genera were differentially abundant between the pre- and post-vasectomy semen samples: an unidentified genus of the family Corynebacteriaceae was increased in pre-vasectomy samples, while *Porphyromonas, Peptoniphilus, Varibaculum*, and *Anaerococcus* were more abundant after the vasectomy. Interestingly, the genera Porphyromonas and Peptoniphilus have previously been associated with bacterial vaginosis, suggesting that semen could be a potential microbial reservoir (Baud *et al.*, 2025).

When urine samples were included into the paired comparative analysis to control for overlap with the urinary microbiome, only *Globicatella*, *Anaerococcus*, and *Escherichia–Shigella* showed relative increase in post-vasectomy semen, however, after the multiple testing correction, no significant differences remained. This suggests that at least part of the apparent microbial differences between the pre- and post-vasectomy semen may be attributable to inter-individual variability or shared taxa between the semen and urine, rather than a direct effect of the vasectomy itself.

When evaluating the genus richness, the vasectomy procedure had an effect of increasing the α-diversity among the seminal samples. In line with our findings, a pioneering study found that only two of the pre-vasectomy samples, but all five of the post-vasectomy samples, tested positive for bacteria (Kiessling *et al.*, 2008). However, other studies with limited sample size detected the contrary and linked vasectomy to a reduced α-diversity in paired and unpaired semen samples (Suarez Arbelaez *et al.*, 2023). Nevertheless, there appears to be consistency in vasectomy affecting the composition and abundance of the seminal microbiome, with the observed patterns likely reflecting dynamic interactions among connected compartments along the male urogenital tract, including the testis and epididymis. Indeed, testis has been shown to harbour microbial sequences (Alfano *et al.*, 2018; Molina *et al.*, 2021). Clearly, this needs further validation, but this rise in bacterial richness may also be linked to the epididymis-unique defensins, which are a group of antimicrobial proteins recognized as vital in response to pathogens (Yamaguchi *et al.*, 2002; Yenugu *et al.*, 2004). Humans are known to produce a large quantity of these defensins in the epididymis, including certain types that are exclusive to this organ (Kiessling *et al.*, 2008). One could hypothesize that following vasectomy, the removal of defensins effect may compromise the local antimicrobial milieu, allowing colonization or overgrowth of bacterial taxa that are normally suppressed, thereby contributing to the increased richness and compositional shift observed in post-vasectomy semen samples. Additionally, interindividual variability in defensin activity may be influenced by genetic variations in defensin genes, such as members of the *DEFB* family (Fernández-Torres *et al.*, 2024). It has been shown that polymorphisms in the promoter area of *DEFB1* associated with impaired defensin expression in the skin and contributed to the persistent Staphylococcus aureus carriage, demonstrating that the host genetic variation could shape microbial colonization patterns (Nurjadi *et al.*, 2013).

Further, β-diversity, an indicator of inter-sample variability, significantly changed after vasectomy, indicating that the semen microbial communities fluctuate after male sterilization; however, the proportion of variance explained was limited, and the inter-individual variation remained the prevailing source of variability. No specific microbial taxa shifts were identified when comparing pre- and post-vasectomy samples, while a significant difference in microbiome functionality predicted from sequencing data was detected, particularly in the fatty acid elongation pathway. Post-vasectomy samples presented a reduction in the lipid metabolism as well as a significant increase in the relative abundance of genera that are not involved in the host lipid metabolism such as *Globicatella*, *Escherichia–Shigella*, and *Anaerococcus*. In fact, *Escherichia–Shigella* has been associated with dysbiosis and LPS-mediated inflammation, which can disrupt lipid metabolism and decrease the production of beneficial metabolites such as short-chain fatty acids (Wang *et al.*, 2016). Pre-vasectomy samples, on the other hand, showed a significant increase in the relative abundance of bacterial taxa involved in lipid metabolism, such as *Cloacibacterium* (Ram *et al.*, 2018). These exploratory analyses suggest that the vasectomy may influence the seminal microenvironment toward a less metabolically active and potentially more pro-inflammatory microbiome. Nevertheless, as the functional profiles were inferred from sequencing data, these findings should be considered hypothesis-generating rather than direct evidence of altered microbial activity and should be confirmed with experimental validation of metabolic pathways and its metabolites together with inflammatory markers.

The critical role of lipid metabolism in male reproductive health has been recognized (Chen *et al.*, 2025), where the capacity to synthesize and modify fatty acids is impaired in men with low semen quality (Guerra-Carvalho *et al.*, 2024). Interestingly, men with azoospermia have also been shown to have reduced lipid metabolism and altered microbiome (Houle *et al.*, 2023). These similarities in the microbial and metabolic alterations in post-vasectomy individuals and men with azoospermia may refer to the role of microbes in seminal functions and underlie similar mechanisms in the seminal microenvironment that may influence male reproductive/genitourinary health (Alfano *et al.*, 2018; Chen *et al.*, 2018).

Our study is the largest using the paired urine and semen samples together with meticulous negative and positive controls throughout the study, nevertheless, there are limitations to highlight. First, the use of marker gene analysis limits the bacterial identification on the species level. Secondly, the absence of absolute bacterial load quantification (e.g. qPCR, dPCR) limits the ability to differentiate true biological changes from compositional shifts. Although we applied stringent contamination controls and in silico decontamination methods following current best-practice recommendations (Davis *et al.*, 2018), future studies incorporating absolute quantification would strengthen the biological interpretation of microbiomes in the low microbial biomass sites such as semen and urine. Additionally, since our study followed a pre-post design without a non-vasectomized control group sampled over the same period, temporal confounders such as seasonal variation and lifestyle changes could have partly accounted for some of the microbiome shifts. Nevertheless, previous studies suggest that the male urogenital microbiome remains relatively stable over the time in the absence of surgical or pharmaceutical perturbations (Mehta *et al.*, 2022). Similarly, large-scale studies on the gut microbiome in men have shown that taxonomic and functional profiles are largely conserved within individuals across time intervals of up to 6 months, with within-person variation substantially lower than between-person differences (Mehta *et al.*, 2018). Additionally, the period of sexual abstinence prior to semen collection inherently minimizes the possibility of very recent microbial exchange between partners, further reducing the chances that such events influenced our results (Baud *et al.*, 2025).

## Conclusions

Our study results highlight that the seminal and urine microbiomes harbour high amounts of contaminants that need to be controlled for. Also, although a big part of the detected microbiomes in the two adjacent sites share the same bacterial communities, there are marked differences in the bacterial compositions between semen and urine samples. The vasectomy procedure seemed to slightly influence these microbiomes, while no specific microbial taxa remained statistically significant after multiple comparison correction. On the other hand, the detected microbial shifts might influence the microbiome functionality profiles, including fatty acid metabolism pathway. Altogether, our study findings provide new insights into the dynamics and composition of the seminal microbiome and suggest that the seminal microbial communities may be influenced by connected genitourinary compartments, where the vasectomy procedure could potentially induce slight changes in the seminal microenvironment. Nevertheless, further longitudinal and mechanistic studies would be required to determine whether the observed microbial shifts have any clinical relevance. Understanding these microbial dynamics is crucial for elucidating factors that influence male reproductive health and may contribute to the future development of novel diagnostic, therapeutic, and reproductive health strategies.

## Supplementary Material

hoag043_Supplementary_Data

## Data Availability

The 16S rRNA gene sequencing data have been uploaded to the SRA database under BioProject ID PRJNA1355064.
