# Supplementary material for: The impact of vasectomy on the seminal microbiome: possible implications and source of microbes
Source: Hum Reprod Open. 2026 May 12;2026(3):hoag043. doi: 10.1093/hropen/hoag043 (PMC13278845; doi:10.1093/hropen/hoag043)
Supplement: hoag043_Supplementary_Data [file hoag043_supplementary_data.zip › Supplementary Figure S1.pdf]

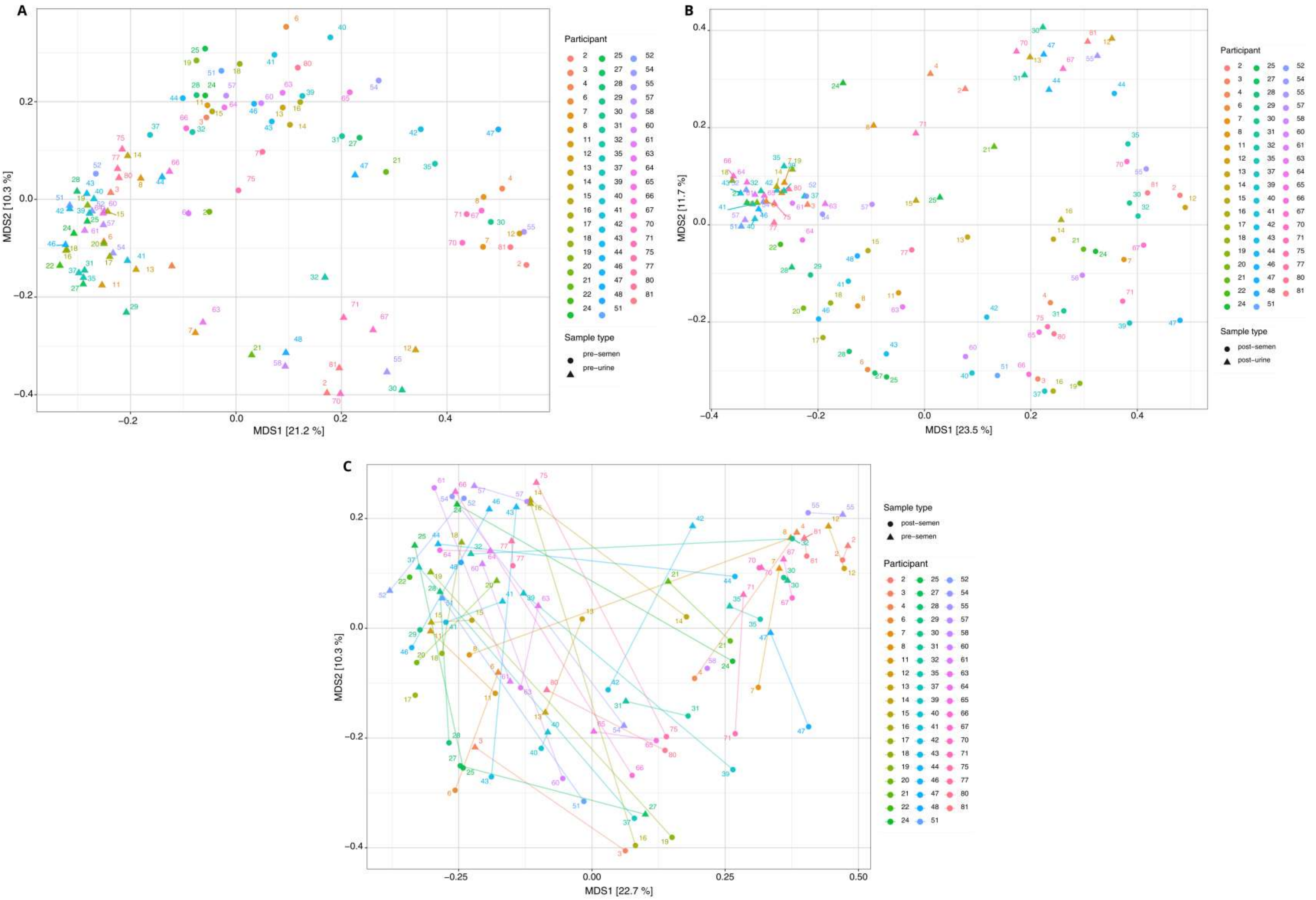
A
MDS1 [21.2 %]
MDS2 [10.3 %]
Participant
Sample type
pre-semen
pre-urine
B
MDS1 [23.5 %]
MDS2 [11.7 %]
Participant
Sample type
post-semen
post-urine
C
MDS1 [22.7 %]
MDS2 [10.3 %]
Sample type
post-semen
pre-semen
Participant

Supplementary Figure S1. A. β-diversity represented by a principal coordinate analysis (PCoA) plot based on the Bray-Curtis distance of patients with paired samples according to sample source: pre-vasectomy urine and semen (PERMANOVA, R^2=0.522, *p*-value=0.063). **B.** β-diversity represented by a principal coordinate analysis (PCoA) plot based on the Bray-Curtis distance of patients with paired samples according to sample source: post-vasectomy urine and semen (PERMANOVA, R^2=0.563, *p*-value=0.001). **C.** β-diversity represented by a principal coordinate analysis (PCoA) plot based on the Bray-Curtis distance of patients with paired samples according to sample source: pre- and post-vasectomy semen (PERMANOVA, R^2=0.679, *p*-value=0.001). Lines connect paired samples from the same individual, illustrating the trajectories between pre- and post-vasectomy.
