# Supplementary material for: The impact of vasectomy on the seminal microbiome: possible implications and source of microbes
Source: Hum Reprod Open. 2026 May 12;2026(3):hoag043. doi: 10.1093/hropen/hoag043 (PMC13278845; doi:10.1093/hropen/hoag043)
Supplement: hoag043_Supplementary_Data [file hoag043_supplementary_data.zip › Supplementary_Figure_S2.pdf]

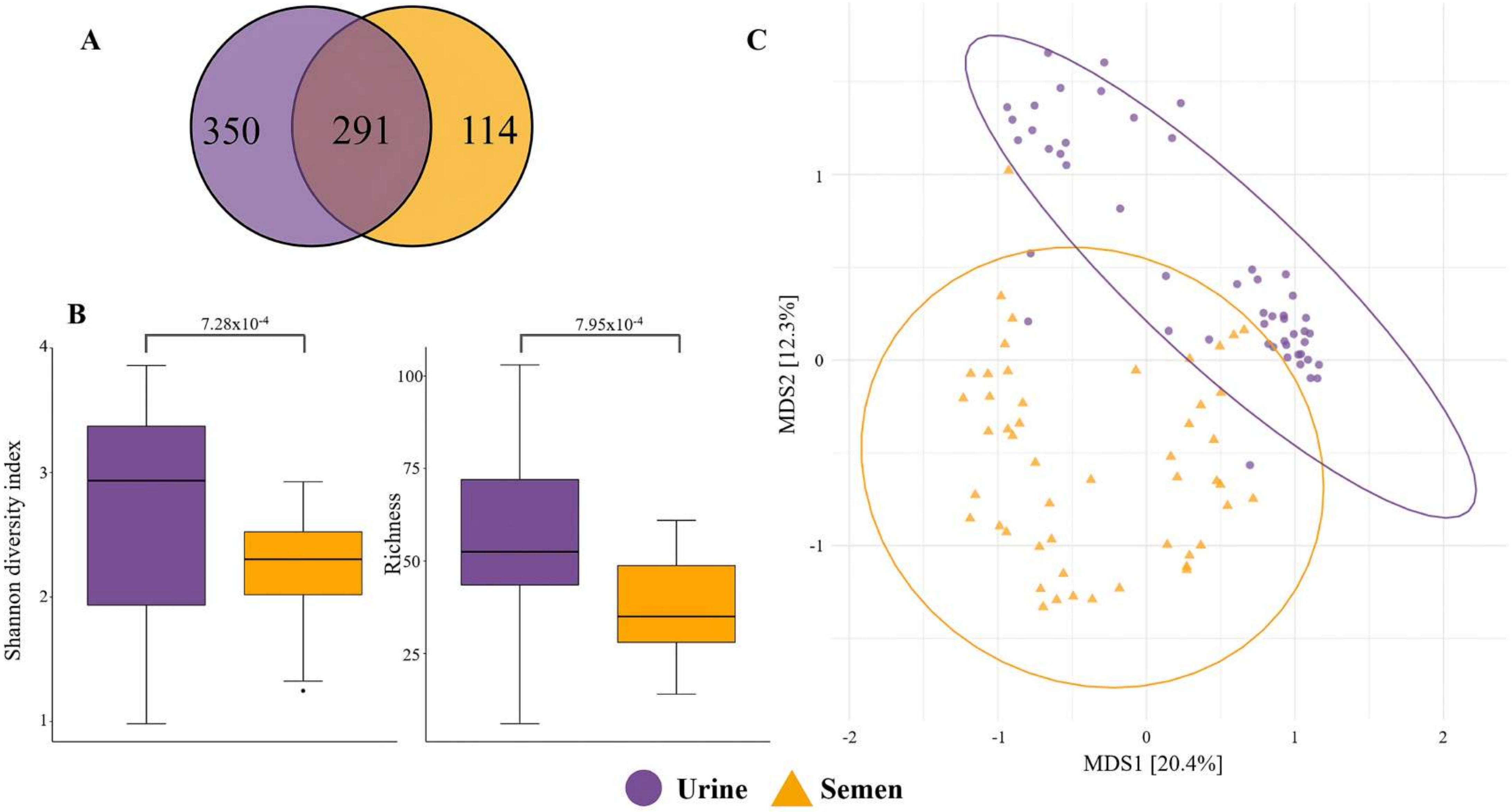
A
350
291
114
B
7.28x10-4
7.95x10-4
Shannon diversity index
Richness
C
MDS2 [12.3%]
MDS1 [20.4%]
Urine
Semen

Supplementary Figure S2. A. Venn diagram showing the distribution of identified genera according to sample source: urine (purple), semen (orange). A total of 755 ASVs were identified. Of these, 350 genera (54.6% of urine ASVs and 46.4% of all ASVs) were exclusively identified in urine samples, while 114 genera (28.1% of semen ASVs and 15.1% of all ASVs) were exclusively related to semen. The remaining 291 genera (38.5% of all ASVs) were shared by both niches. **B.** α-diversity evaluated by Shannon diversity index (left) and observed richness (right). **C.** β-diversity represented by a principal coordinate analysis (PCoA) plot based on the Bray-Curtis distance.
